# Automatic Micro-Expression Analysis: Open Challenges

**DOI:** 10.3389/fpsyg.2019.01833

**Published:** 2019-08-07

**Authors:** Guoying Zhao, Xiaobai Li

**Affiliations:** ^1^School of Information and Technology, Northwest University, Xi'an, China; ^2^Center for Machine Vision and Signal Analysis, University of Oulu, Oulu, Finland

**Keywords:** micro-expression, facial expression, automatic analysis, computer vision, machine learning

Micro-expressions, the fleeting and involuntary facial expression, often occurring in high-stake situations when people try to conceal or mask their true feelings, became well-known since 1960s, from the work of Haggard and Isaacs (1966) in which micro-expression was firstly termed as micromomentary facial expressions, and later from the work of Ekman and Friesen (1969).

Micro-expressions are too short (1/25 to 1/2 s) and subtle for human eyes to perceive. Study (Ekman, 2002) shows that for micro-expression recognition tasks, ordinary people without training only perform slightly better than chance on average. So computer vision and machine learning methods for automatic micro-expression analysis become appealing. Pfister et al. (2011) started pioneering research on spontaneous micro-expression recognition with the first publically available spontaneous micro-expression dataset: SMIC, and achieved very promising results that compare favorably with the human accuracy. Since then micro-expression study in computer vision field has been attracting attentions from more and more researchers. A number of works have been contributing to the automatic micro-expression analysis from the aspects of new datasets collection (from emotion level annotation to action unit level annotation; Li et al., 2013; Davison et al., 2018), micro-expression recognition (from signal apex frame recognition to whole video recognition; Wang et al., 2015; Liu et al., 2016; Li Y. et al., 2018; Huang et al., 2019) and micro-expression detection (from micro-expression peak detection to micro-expression onset and offset detection; Patel et al., 2015; Xia et al., 2016; Jain et al., 2018). First completed system integrating micro-expression recognition and detection toward reading hidden emotions (Li X. et al., 2018) has been reported by MIT Technology Review (2015) and achieved increasing attention, in which the machine learning method obtained 80.28% for three class (positive/negative/surprise) recognition for 71 micro-expression video clips recorded from eight subjects and 57.49% for five class (happiness, disgust, surprise, repression, and other) recognition for 247 micro-expression video clips recorded from 26 subjects (Li X. et al., 2018), which has outperformed the recognition capability of human subjects (Li X. et al., 2018).

However, there are still many open challenges which need to be considered in the future research. Several main challenges related with micro-expression study are discussed in details in the following.

## Datasets

Data are a central part in micro-expression research. Even though there have been more datasets collected and released, from the first SMIC (Li et al., 2013), to CASME (Yan et al., 2013), CASME II (Yan et al., 2014), SAMM (Davison et al., 2018), MEVIEW dataset (Husak et al., 2017), and CAS(ME)^2^ (Qu et al., 2018), including more subjects, higher resolution, and more videos, the scale of current datasets is just hundreds of micro-expression videos captured from 30 to 40 subjects, and there still lacks high quality, naturally collected and well-annotated large scale micro-expression data captured by different sensors for training efficient deep learning methods, which is a big obstacle for the research. As inducing and labeling micro-expression data from scratch is extremely challenging and time consuming, it is not feasible for any single research group to gather data scale of larger than tens of thousands of samples. One possible option for future micro-expression data construction work could be utilizing the vast source of YouTube videos and mining with some video tagging techniques for candidate clips then follow with human labeling. Another option could be collaborative and parallel data collection and labeling through cloud sourcing.

Moreover, one potential application of micro-expression analysis is lie detection. When lying, more contradictory behaviors could be found in verbal and non-verbal signals (Navarro and Karlins, 2008), perhaps more micro-expressions could appear. Therefore, new datasets containing not only facial expression and micro-expression, but also audio speech could be beneficial for micro-expression study.

## Action Units Detection of Micro-Expressions

Facial Action Coding System (FACS) is an anatomically based system for measuring facial movements (Ekman and Friesen, 1978), which is used to describe visually distinguishable facial activity on the basis of many unique action units (AUs). In most of the previous work (Wang et al., 2015; Li X. et al., 2018), micro-expressions were recognized from the whole face without action unit study, and only positive and negative micro-expressions, or limited number of micro-expressions were classified. Instead of directly recognizing a certain number of prototypical expressions as in most of the previous research, AUs can provide an intermediate meaningful abstraction of facial expressions, and carry lots of information which can help better detect and understand people's feelings. Even though AU detection has been taken into consideration for macro-expression analysis (Zhao et al., 2016, 2018; Han et al., 2018; Zhang et al., 2018), including pain detection and pain intensity estimation (Prkachin and Solomon, 2008; Lucey et al., 2011), rare work has been done for AUs in micro-expressions. Future study could pay more attention to explore the relationship between AUs and micro-expressions. For example: is there fixed mapping between the onset of a certain AU (or a sequence of AU combinations) and one micro-expression category, just like the criteria for AU and facial expression correspondence listed in FACS manual? The category of concerned micro-expression emotions is not necessarily limited to the prototypical basic emotions, i.e., happiness, sadness, surprise, anger, disgust and fear, but could also consider other emotions which are out of the above mentioned basic emotion scope, yet very useful for real-world applications, like nervousness, disagreement and contempt. Besides, except those most common emotional AUs (that are considered to be closely related with emotional expressions), e.g., AU1, AU4, and AU12, other AUs which were formally considered as “irrelevant to emotions” also worth more exploration, as studies found that some (e.g., eye blinks and eye gaze change) are employed as disguise behaviors to cover true feelings thus frequently occur WITH the onset of micro-expressions.

## Realistic Situations

Most of the existing efforts on micro-expression analysis have been made to classify the basic micro-expressions collected in highly controlled environments, e.g., from frontal view (without view changes), with stable and bright lighting conditions (without illumination variations), whole face visible (without occlusion). Such conditions are very difficult to reproduce in real-world applications and tools trained on such data usually do not generalize well to natural recordings made in unconstrained settings. Effective algorithms for recognizing naturally occurring micro-expressions which are robust to realistic situations with the capability to deal with pose changes, illumination variations and poor quality of videos, recorded in-the-wild environment must be developed.

## Macro- and Micro- Expressions

Previous work about facial expression has concerned with either micro- or macro-expressions. For most early micro-expression works, it has been assumed that there are just micro-expressions in a video clip. For example, in the collection of most micro-expression datasets (Li et al., 2013; Yan et al., 2013, 2014; Davison et al., 2018; Qu et al., 2018), subjects were asked to try their best to keep a neutral face when watching emotional movie clips. In this way, the conflict of felt emotion elicited by the movie clip and the strong intention to suppress any facial expression could induce micro-expressions. The consequence in the collected videos is that, if there is micro-expression in the recorded video, it is unlikely to have other natural facial expressions. But in most cases in real life, this is not true. Micro-expressions can appear when there is a macro-expression as well, for example, when people smile, they might furrow forehead very quickly and shortly, which show their true feeling (Ekman and Friesen, 1969). Future studies could also concern the relationship of macro and micro-expressions, and explore methods that can detect and distinguish these two when they co-occur or even overlap with each other in one scenario, which would be very helpful to understand people's feelings and intentions more accurately.

## Context Clues and Multi-Modality Learning

In social interactions, people interpret other's emotions and situations based on many things (Huang et al., 2018): people in the interaction, their speech, facial expression, cloths, body pose, gender, age, surrounding environments, social parameters, and so on. All these can be considered as contextual information. Some people are better emotion readers, as they can sense others' emotion more accurately than the rest. These people usually pick up subtle clues from multiple aspects, not only the facial expressions (Navarro and Karlins, 2008). One original motivation for the study of micro-expression is to explore people's suppressed and concealed emotions, but we shouldn't forget that micro-expression is only one of the many clues for such purpose. Future studies should try broaden the scope and consider combining micro-expression with other contextual behaviors, e.g., eye blink, eye gaze change, hand gesture change, or even whole body posture, in order to achieve better understanding of people's hidden emotions on a fuller scope.

Recent psychological research demonstrates that emotions are a multimodal procedure which can be expressed in various ways. “Visual scenes, voices, bodies, other faces, cultural orientation, and even words shape how emotions is perceived in a face” (Barrett et al., 2011). As well emotional data can be recorded with different sensors, e.g., color camera, near infrared camera, depth camera, or physiological sensors, for recording emotional behaviors or bodily changes. This also applies to the study of micro-expression and suppressed or hidden emotion. One single modality could be unreliable, as one certain behavior pattern could be just related to physiological uncomfort or personal habit, but has nothing to do with emotional states. So only when multiple cues are considered together we could achieve more reliable emotion recognition. There is very little investigation in this respect so far, and future micro-expression studies could consider combining multi-modality data for micro-expression and hidden emotion recognition.

## Analysis for Multiple Persons in Interactions

The current micro-expressions research focuses on single person watching affective movies or advertisements, which is reasonable in the early stage for making challenging tasks easier and more feasible. Later it is surely that the research will be shifting toward more realistic and challenging interaction environments where multiple persons are involved. Natural interactions will induce more natural and spontaneous emotional responses in terms of facial expressions and micro-expressions, but the scenario will also become very complicated. It would be very interesting to explore not only individual level of emotional changes, but also the interpersonal co-occurrence (e.g., mimicry or contagion), and the affective dynamics of the whole group.

## Discussion

We have discussed the progress and the open challenges in automatic micro-expression analysis. Solving these issues needs interdisciplinary expertise. The collaboration of machine learning, psychology, cognition and social behavior is necessary for advancing the in-depth investigation of micro-expressions and related applications in real world.

## Author Contributions

All authors listed have made a substantial, direct and intellectual contribution to the work, and approved it for publication.

### Conflict of Interest Statement

The authors declare that the research was conducted in the absence of any commercial or financial relationships that could be construed as a potential conflict of interest.
